# Gender influences resident physicians’ perception of an employee-to-employee recognition program: a mixed methods study

**DOI:** 10.1186/s12909-024-05083-0

**Published:** 2024-02-01

**Authors:** Jessica S. Tischendorf, Laura K. Krecko, Rachel Filipiak, Fauzia Osman, Amy B. Zelenski

**Affiliations:** 1 Division of Infectious Disease, Department of Medicine, University of Wisconsin School of Medicine and Public Health, Medical Foundation Centennial Building Room 5263, 1685 Highland Avenue, Madison, WI 53705 USA; 2 Department of Surgery, University of Wisconsin School of Medicine and Public Health, 600 Highland Avenue, Madison, WI 53792 USA; 3 Department of Medicine, University of Wisconsin School of Medicine and Public Health, 1685 Highland Avenue, Madison, WI 53705 USA

**Keywords:** Graduate medical education, Gender bias, Interprofessional, Employee recognition

## Abstract

**Background:**

Burnout is prevalent in medical training. While some institutions have implemented employee-to-employee recognition programs to promote wellness, it is not known how such programs are perceived by resident physicians, or if the experience differs among residents of different genders.

**Methods:**

We used convergent mixed methods to characterize how residents in internal medicine (IM), pediatrics, and general surgery programs experience our employee-to-employee recognition ("Hi-5″) program. We collected Hi-5s received by residents in these programs from January 1, 2021–December 31, 2021 and coded them for recipient discipline, sex, and PGY level and sender discipline and professional role. We conducted virtual focus groups with residents in each training program.

**Main measures and approach:**

We compared Hi-5 receipt between male and female residents; overall and from individual professions. We submitted focus group transcripts to content analysis with codes generated iteratively and emergent themes identified through consensus coding.

**Results:**

Over a 12-month period, residents received 382 Hi-5s. There was no significant difference in receipt of Hi-5s by male and female residents.

Five IM, 3 surgery, and 12 pediatric residents participated in focus groups. Residents felt Hi-5s were useful for interprofessional feedback and to mitigate burnout. Residents who identified as women shared concerns about differing expectations of professional behavior and communication based on gender, a fear of backlash when behavior does not align with gender stereotypes, and professional misidentification.

**Conclusions:**

The “Hi-5” program is valuable for interprofessional feedback and promotion of well-being but is experienced differently by men and women residents. This limitation of employee-to-employee recognition should be considered when designing equitable programming to promote well-being and recognition.

**Supplementary Information:**

The online version contains supplementary material available at 10.1186/s12909-024-05083-0.

## Introduction

Resident physicians across medical and surgical specialties report low well-being and burnout at high rates, impacting women trainees disproportionately [1–3]. Strategies to mitigate burnout and promote wellness during medical training are urgently needed. As such, institutions have implemented various interventions attempting to improve employee recognition and well-being.

One method posited to increase employee satisfaction is direct employee-to-employee recognition programs, which have been adopted in non-medical and medical fields to improve morale and promote interprofessional collegiality [4–6]. Despite adoption of these programs in healthcare, their impact on residents is not well characterized. Moreover, it remains unknown whether these programs have a differential impact on residents of different genders. Given women trainees experience more discrimination in the workplace [7–11], bias in assessment [12], less afforded autonomy [13, 14], and lower frequency of recognition [15–17], a difference may plausibly exist in how these employee-to-employee programs are experienced by residents of different genders. This represents an area in need of further evaluation.

We used convergent mixed methods to characterize how residents in internal medicine, pediatric, and general surgery residency programs at a single center experience our institution’s employee recognition ("Hi-5″) program.

## Methods

### Hi-5 description

**Fig. 1:**
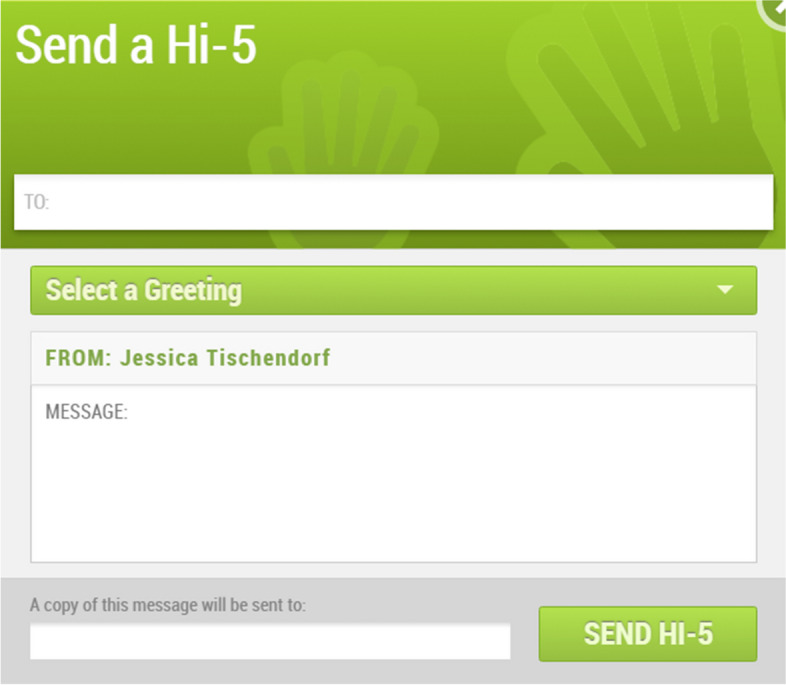
Hi-5 submission form

The “Hi-5 Program” is a well-established direct employee-to-employee electronic recognition program used at our institution (Fig. 1: Hi-5 submission form). This platform allows employees to send an email (a “Hi-5”) via an online submission form to any other employee in our health system. The intent is to recognize and/or thank peers for their work. A Hi-5 is sent to the employee being recognized, as well as to that employee’s immediate supervisors. For residents at our institution, their Graduate Medical Education (GME) program director and program coordinator are automatically notified of receipt of a Hi-5 and can view the sender, receiver, and content of the Hi-5.

### Participants and setting

We prospectively collected Hi-5s received by residents in the internal medicine, pediatric, and general surgery residency programs at our institution from January 1, 2021 – December 31, 2021. These are mid-sized residency programs situated within a tertiary care academic medical center in the Midwest. In 2020-2021 and 2021-2022, residency class sizes were 88 and 89 for internal medicine, 48 and 47 for pediatrics, and 57 and 55 for general surgery respectively. We obtained resident rosters from residency program coordinators, which provided training year and sex as indicated by the resident in their official training record. In the internal medicine residency, females comprised 45% of the class size in both 2020-2021 and 2021-2022; in pediatrics, females compromised 77% of the 2020-2021 class and 66% of the 2021-2022 class; in general surgery, females accounted for 48% and 53%. Regarding language used to describe sex and gender; our quantitative data was based on training records requiring identification of “male” or “female” sex, hence, our quantitative data is discussed with sex terms. The lack of ability to represent diverse genders in our quantitative data beyond binary male/female is a limitation of this study. Participants in focus groups were asked to self-identify gender, therefore, qualitative results and interpretation are presented using gender terms. Acknowledging the evolving understanding of gender identity, we will use man/woman gender identifiers when referencing relevant literature even when male/female were used by authors.

Our work was approved by the University of Wisconsin-Madison Health Sciences Institutional Review Board.

### Data collection

Program coordinators from participating residencies forwarded Hi-5 email notifications to a single study team member (R.F.) who de-identified the Hi-5 prior to data analysis. During the de-identification process, names of recipients were removed and each Hi-5 was coded for recipient training program, gender and post-graduate year (PGY) level, as well as sender discipline (when one was specified in the employee directory) and professional role (nurse, physician, pharmacist, etc.). We initially sought to infer sender gender from available employee photographs but given inability to triangulate with another source do not present this in our analysis.

### Data analysis

All baseline categorical data were summarized as counts and percentages. We compared all groups using a chi-squared test or a Fisher’s exact test for values less than 5. *P*-values less than or equal to 0.05 were considered significant. All analyses were conducted using STATA version 17. To include all Hi-5s regardless of sender role, even those with low volume of Hi-5s, we combined those with a similar nature of interactions with residents. The sender role designations we used for Hi-5 analysis were: (1) attending physician, (2) chief resident, (3) trainee (medical student, resident or fellow), (4) nurse, (5) other allied health professional (physical therapy, occupational therapy, nurse practitioner, physician assistant, pharmacist, nurse anesthetist, surgical technician, respiratory therapist, child life specialist, social worker and fitness supervisor), and (6) administrative (quality improvement specialist, clinical documentation specialist, GME coordinator, improvement advisor, organ allocation specialist, emergency department coordinator, patient scheduler).

### Focus groups

To understand how residents experience the Hi-5 program, we conducted three separate focus groups on a secure video platform with residents in internal medicine, pediatrics and general surgery. We developed a semi-structured focus group guide through an iterative process among three study team members (J.T., L.K., A.Z.). This focus group guide explored participants’ Hi-5 sending and receiving practices, motivators for sending and receiving Hi-5s, perceived personal and professional impact of receiving Hi-5s, and the perceived influence of gender on their experience of the Hi-5 program. We presented our semi-structured interview guide for review at a University of Wisconsin School of Medicine and Public Health (UWSMPH) Qualitative Research Group meeting, which provided key feedback to inform our guide. We piloted our focus group guide with four internal medicine chief residents and incorporated their feedback on question sequence and structure into the final script.

We recruited participants primarily via institutional email, with three reminders sent through program coordinators to residency listservs. A brief announcement was also made by study personnel at regularly scheduled didactics for the internal medicine and pediatric residencies. For internal medicine and pediatric participants, focus groups were conducted during regularly scheduled education time. The general surgery focus group was scheduled based on participant availability and took place outside of regularly scheduled education time. Participants of all genders were invited to participate. To avoid the possible inhibition of responses that a peer or supervisor facilitating a focus group in their own department might introduce, we selected study personnel with no routine clinical or educational contact with a given residency to facilitate.

Verbal consent from participants was obtained prior to the start of each focus group. Focus groups were recorded and transcripts were generated automatically by the virtual meeting platform. Study personnel edited the automatically generated transcripts for accuracy by listening to and observing video recordings. We de-identified the transcripts and assigned each participant a code indicating their self-identified gender and PGY level. De-identified transcripts were submitted for qualitative analysis.

### Qualitative analysis

We used content analysis to analyze the focus group transcripts. After initial codes were generated, these were reviewed iteratively by three study team members through consensus coding (JS, LK, AZ) to collate emergent themes. All qualitative data were co-coded for gender of the participant to allow for later gender-based analysis. Illustrative participant quotes were bookmarked during qualitative data analysis. Once emergent themes were identified, we offered the opportunity for member checks with residents from each training program to ensure our interpretation reflected their experience. All qualitative data was managed using NVIVO software (QSR International Pty Ltd. Version 12, 2018). Codes were generated and qualitative data analyzed through the lens of role congruity theory [18] and by applying established principles of high-quality feedback [19]. Included quotes were lightly edited for readability (e.g., removed filler words).

## Results

### Hi-5 receipt

Over a 12-month period, 382 Hi-5s were sent to 211 residents in the internal medicine, general surgery, and pediatric training programs, with the majority sent to internal medicine residents (196), followed by surgery (110) and pediatrics (76). Nurses were the most common senders, sending 31.9% of Hi-5s, followed by chief residents and attending physicians sending 19% of those received by residents. Residents received Hi-5s from other trainees 18% of the time (Table 1).

**Table 1:** Sender and receiver characteristics of Hi-5s sent to pediatrics, IM and surgery residents. Counts are of Hi-5s received; percents represent proportion of Hi-5s received within each category, as either a proportion of the total (column 1), or of those received by female (column 2) or male (column 3) residents. In 2020-2021 and 2021-2022, residency class sizes were 88 (45% female) and 89 (45% female) for IM, 48 (77% female) and 47 (66% female) for pediatrics, and 57 (48% female) and 55 (53% female) for general surgery, respectively

Variable	Total Hi-5s *N* = 382	Hi-5s received by female residents *N* = 199	Hi-5s received by male residents *N* = 183
Receiver Discipline			
IM	196 (51.3)	99 (49.7)	97 (53.0)
Surgery	110 (28.8)	45 (22.6)	65 (35.5)
Peds	76 (19.9)	55 (27.6)	21 (11.5)
Academic year			
2020 - 2021	211 (55.2)	117 (58.8)	94 (51.4)
2021 - 2022	171 (44.8)	82 (41.2)	89 (48.6)
Receiver PGY			
PGY1	115 (30.1)	59 (29.6)	56 (30.6)
PGY2	137 (35.9)	66 (33.2)	71 (38.8)
PGY3	99 (25.9)	56 (28.1)	43 (23.6)
PGY4	17 (4.5)	12 (6.0)	5 (2.7)
PGY5	8 (2.1)	1 (0.5)	7 (3.8)
Research	6 (1.6)	5 (2.5)	1 (0.5)
Sender Role			
Admin	14 (3.7)	11 (5.5)	4 (2.2)
Allied health professionals, other	30 (7.9)	13 (6.5)	16 (8.7)
Attending	74 (19.4)	41 (20.6)	33 (18.0)
Chief Resident	73 (19.1)	36 (18.1)	37 (20.2)
Nurse	122 (31.9)	55 (27.6)	67 (36.6)
Trainee	69 (18.1)	43 (21.6)	26 (14.2)

Across training programs, there was no statistical difference in receipt of Hi-5s by male and female residents, though in the latter half of our study period, there was a non-significant trend toward males receiving more Hi-5s from nurses, attending physicians, chief residents and allied health professionals; while females tended to receive more Hi-5s from co-trainees and administrative personnel (Table 2). These interactions were less robust when considering training programs in isolation, with one exception: Female general surgery PGY2s received significantly fewer Hi-5s than their male PGY2 colleagues. Program level data is available in supplementary Tables 1, 2 and 3.

**Table 2:** Differences in Hi5 receipt between male and female residents, by sender role. Counts are of Hi-5s received, percents represent proportion of Hi-5s received by male and female residents from respective sender groups

2020–2021												
Sender role	GME residents (total) *N* = 211			Internal medicine residents *N* = 89			Pediatrics residents *N* = 63			Surgery residents *N* = 59		
	Male *N* = 94	Female *N* = 117	*P*	Male *N* = 42	Female *N* = 47	*P*	Male *N* = 15	Female *N* = 48	*P*	Male *N* = 37	Female *N* = 22	*P*
Attending	16 (34.8)	30 (65.2)	0.15	7 (43.8)	9 (56.3)	1.0	1 (8.3)	11 (91.7)	0.43	8 (44.4)	10 (55.6)	0.61
Chief resident	18 (43.9)	23 (56.1)	0.77	17 (44.7)	21 (55.3)	1.0	1 (33.3)	2 (66.7)	0.56	-	-	-
Trainee	17 (43.6)	22 (56.4)	0.75	6 (40)	9 (60.0)	0.78	5 (31.3)	11 (68.8)	0.52	6 (62.7)	2 (25)	0.28
Nurse	31 (51.7)	29 (48.3)	0.47	8 (57.1)	6 (42.9)	0.57	7 (30.4)	16 (69.6)	0.57	16 (69.6)	7 (30.4)	0.16
Allied Health	9 (52.9)	8 (47.1)	0.62	3 (100)	0 (0)	0.11	1 (12.5)	7 (87.5)	0.67	5 (83.3)	1 (16.7)	0.21
Admin	3 (37.5)	5 (62.5)	0.73	1 (33.3)	2 (66.7)	1.0	0 (0)	1 (100)	1.0	2 (50)	2 (50)	1.0
2021–2022												
	GME residents (total) *N* = 171			Internal medicine residents *N* = 107			Pediatrics residents *N* = 13			Surgery residents *N* = 51		
	Male *N* = 89	Female *N* = 82	*P*	Male *N* = 55	Female *N* = 52	*P*	Male *N* = 6	Female *N* = 7	*P*	Male *N* = 28	Female *N* = 23	*P*
Attending	17 (60.7)	11 (39.3)	0.16	11 (64.7)	6 (35.3)	0.60	0 (0)	1 (100)	1.0	6 (60.0)	4 (40.0)	0.51
Chief resident	19 (59.4)	13 (40.6)	0.18	19 (59.4)	13 (40.6)	0.84	-	-		-	-	-
Trainee	9 (30.0)	21 (70.0)	0.17	6 (28.6)	15 (71.4)	0.05	3 (42.9)	4 (57.1)	0.36	0 (0)	2 (100)	0.50
Nurse	36 (58.1)	26 (41.9)	0.08	16 (53.3)	14 (46.7)	1.0	1 (50)	1 (50)	0.43	19 (63.3)	11 (36.7)	0.18
Allied Health	7 (58.3)	5 (41.7)	0.39	2 (50.0)	2 (50)	1.0	2 (66.7)	1 (33.3)	0.16	3 (60.0)	2 (40.0)	0.67
Admin	1 (14.3)	6 (85.7)	0.14	1 (33.3)	2 (66.7)	0.59	-	-	-	0 (0)	4 (100)	0.12

### Focus group themes

**Fig. 2:**
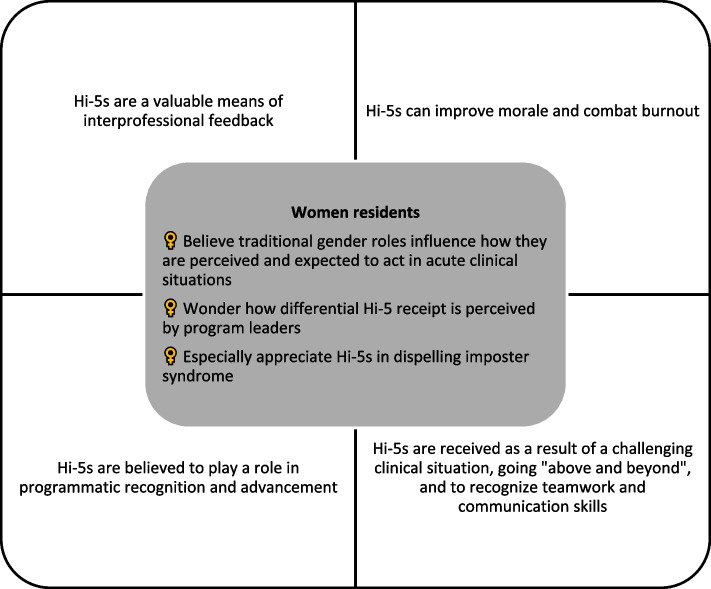
Focus group themes

Focus groups were of mixed gender as self-identified by participants, with 5 participants in the internal medicine group, 12 in pediatrics, and 3 in surgery. No participants self-identified as other than man or woman. Themes from focus group discussions centered on scenarios prompting the receipt of Hi-5s and on the role Hi-5s play in interprofessional feedback, improving morale and self-image, and resident recognition and advancement. In discussing these themes, we prompted participants to consider how resident gender may influence experiences with the Hi-5 program (Fig. 2: Focus group themes).

#### Scenarios prompting receipt of Hi-5s

Residents described typical scenarios in which they would receive a Hi-5; these were largely grouped into (1) challenging clinical situations (“crisis”), (2) instances when the resident was perceived as going “above and beyond” for patient care, and (3) examples of high-quality teamwork and communication skills.

Challenging clinical situations

Residents described high-acuity clinical situations that have led to receipt of a Hi-5. Residents discussed how gender impacts their experiences in these scenarios.

“ … I feel like [it] happens after some kind of difficult situation, whether it’s a code or a difficult patient or a really stressful overnight shift.”

When discussing how challenging clinical scenarios were often a motivator for receiving or sending a Hi-5, several women participants brought up their occasionally experienced tension when asserting themselves as leaders in acute situations.

“I think there’s also a difference in the way that society perceives a woman who is direct versus a man who is direct. Speaking from someone who’s going into cardiology where you’re a team leader, and you need to be direct about what is happening, you know, when you need to get an EKG, you need to get an EKG, you don’t need to go, ‘Well, I mean, I’m a little concerned about the rhythm, so I think probably the next step is the EKG.’ But in general … society looks toward a man who’s direct and goes,’Man, he really just knows what’s up, he knows what he wants’ and then the woman who's direct is ‘bossy’ or worse and so I think that um, it’s hard for us [women] to get Hi-5s for having excellent communication and being direct when we’re perceived poorly from society’s standpoint for doing that.” – Internal medicine resident.

##### Going “above and beyond”

Some residents received Hi-5s when they were perceived as having gone “above and beyond” their typical job duties. Residents discussed how traditional gender roles could alter the expectation of residents and what qualifies as “above and beyond” for men and women residents. One resident, in response to a male colleague describing reluctance to return to a patient’s bedside after several visits that day, stated:

“I didn’t even think about that … assumptions about what different people should be willing and able to do and how it may be more acceptable for a male provider to delay or kind of not be interested in doing the communication piece […]”- Internal medicine resident.

##### Recognizing high quality teamwork and communication

Residents reported that Hi-5s from non-physician colleagues often referenced high-quality teamwork and communication skills.

“I remember it as an intern, if you’re trying like, a new strategy of talking to a family, or, how you’re filling in a nurse … if you happen to get a Hi-5 or good feedback from that, it’s like, ‘well, that worked’.” – Pediatrics resident.

However, several women residents shared concerns that differing expectations of men and women residents may impact the likelihood of receiving Hi-5s from their non-physician colleagues.

“I think we always wonder as female residents, like, there’s this unspokenness [sic] that you have to be not only a really good physician, but you have to be really nice and you have to be friends with all the nurses, and you have to go out of your way to be helpful to everyone, even in things that are not your scope of practice.” – General surgery resident.

“[…] historically, surgeons are supposed to be like these mean people [who are] hard to get along with. So, if he’s like, this male surgeon, who’s nice, then people are like, ‘Oh, well, send them a Hi-5,’ but he should be acting that way anyways.” – Pediatrics resident.

“Or we’re not recognized as being physicians point blank, like being assumed that we are nursing staff or other care providers.” – Internal medicine resident.

#### Hi-5s as a means of interprofessional feedback

Residents are accustomed to receiving formal feedback from attending physicians but recognized a paucity of interprofessional feedback. Hi-5s help to fill this gap; residents felt that recognition via Hi-5s from other professions helps to reinforce behaviors valued by these colleagues.

“The Hi-5s are nice too, because it actually usually does point out to me, like, different behaviors that I did that people actually do find nice even if at the time I didn’t realize how big of a deal it meant to the nursing staff, for example, or social work, so then I, [it] kind of helps me know to do that in the future.” – Internal medicine resident.

Residents also highlighted the strengths of Hi-5s as a feedback mechanism. In contrast to the occasionally vague and delayed feedback received through formal evaluation processes at the conclusion of resident rotations, Hi-5s provide specific feedback with temporal proximity to a relevant clinical scenario or behavior.

“[…] I also like the very specific aspect of it too. It’s not like, when you receive feedback that’s like, “Read more” or “Your medical knowledge is excellent” or something, it’s like, very specific concrete feedback, which is always fantastic, too.” – Internal medicine resident.

#### Hi-5s as a means of improving morale and self-image

Overall, residents described the experience of receiving Hi-5s very positively. The unexpected nature of the praise was often referred to as a highlight of their clinical work and was viewed to mitigate burnout. Knowing the sender went outside their usual workflow to recognize them was impactful.

“I love getting Hi-5s – it’s just such a little silver lining … there are a few times on wards or all of a sudden I would see a Hi-5 show up in my email, and it was just like, just a little extra boost that I needed to get to the end of that, whatever stretch that I was on.” – Pediatric resident

Women residents specifically referenced the role of Hi-5s in combatting a sense of imposter syndrome.

“ … there’s plenty of times, especially as a trainee, where I feel like, I am not doing the job, or I am failing at the job that I’m trying to do, especially when it’s like a difficult situation with a worsening patient on the floor or something like that […], when I’ve received a Hi5 within the next few days after, it does really dispel that thought of imposter syndrome…” – Internal Medicine resident.

One woman resident shared her experience after receiving a Hi-5 following a challenging call shift in the intensive care unit.

“It actually made me cry because I was so worried that I think it really helped to dispel some imposter syndrome. Like oh, what was that night? It was terrible. I was also sleep deprived when I read it. But it just made such a big difference in my confidence.” – Internal Medicine resident.

#### Implication of Hi-5s on professional recognition and advancement

Residents believed Hi-5s carry significant professional impact. They perceived them to be used by program leadership to explicitly or implicitly support future letters of recommendation, substantiate receipt of training program awards, factor into chief resident selection, and distinguish fellowship and job applicants of otherwise similar caliber.

“I really like the fact that it does go to our program leadership and oftentimes they will then send you an email to, like kind of reinforcing whatever the Hi-5 said. And I think that’s nice because they’re hardly ever with us on wards … So, it’s like, kind of an eye into what we’re doing and I think that’s nice for them to know just like when we have our reviews and things like that ….” – Internal medicine resident.

A woman resident raised concern about how possible differential receipt of Hi-5s can disadvantage some residents in the eyes of their program leadership.

“If you see certain people, like getting a Hi-5 every week you assume they’re doing a very good job and if he [program director] sees into somebody’s never got the Hi-5 you probably, you might think they’re not doing as good of a job, so I can see how that’d be a problem.” – General surgery resident.

## Discussion

Our institution’s employee-to-employee recognition program, the “Hi-5” program, is valuable for interprofessional feedback and promotion of well-being, but may be experienced differently by men and women residents in internal medicine, pediatrics, and general surgery.

Overall, there was no difference in the number of Hi-5s received by male and female residents in our study. However, in the latter half of our study period, which spans the first six months of the academic year, there was a non-significant tendency for male residents to receive more Hi-5s from interprofessional members of the clinical team and attending physicians. While our single-center study did not demonstrate a statistically significant difference in Hi-5 receipt, previous work has shown more favorable assessments of men residents by nurses than women residents [20, 21], a perception shared by women residents in our focus groups. The perception of differential treatment by interprofessional clinical team members is well described by women physicians [10, 11, 22, 23] and is discordant with studies suggesting better outcomes for patients cared for by women physicians [24, 25]. The explanation for these observations is complex and contributes to the tension some women physicians experience when deviating from their traditional gender role in the clinical setting [18, 26, 27].

We hypothesize that women trainees could be disadvantaged in each of the major scenarios in which Hi-5s were received in our study. Traditional gender roles condition women to display more communal traits: pertinent to the present study – egalitarian, good communicators and team players. When women fail to align with these societal expectations, the dissonance can lead to backlash against them, making it less likely that they are recognized for being effective leaders in crisis situations [18, 28]. The expectation for strong communication skills for women in the clinical setting could also contribute to lack of recognition for these attributes compared to their men colleagues, for whom strong communication may be viewed as “above and beyond”, as these skills do not align with their traditional gender role. Professional role misidentification, which occurs more often for women residents, may also contribute to less recognition from those who work with residents clinically [9, 29–31].

Residents highlighted Hi-5s as a valuable means of specific and timely feedback, characteristics often lacking in formal evaluations [32, 33]. Should women residents receive fewer Hi-5s from their interprofessional clinical team members, it may exacerbate previously described deficiencies in feedback they receive [34].

Differential receipt of Hi-5s from clinical colleagues may limit the effect of the Hi-5 program on well-being for women residents. With studies showing that women residents are more likely to experience burnout [2, 3, 35, 36], being aware of the limitations of an employee-to-employee recognition program in mitigating this distress is key. Further, Hi-5s were perceived as a way to dispel imposter syndrome, which is more often experienced by women trainees [37]. Stereotype threat, the fear of confirming a negative stereotype associated with a group one belongs to [38], is a known contributor to poor psychological health among women residents [39]. Ensuring that women trainees receive affirmation in other venues if not receiving it as often in the clinical setting can mitigate stereotype threat and improve performance [40].

While we did not assess the nature of Hi-5 use by program leadership in advancement and recognition in each residency program, the general understanding by residents that Hi-5s are used in advancement decisions is concerning. If gender influences the likelihood that a resident receives a Hi-5, from whom, or under which circumstances, the concern for how that differential receipt could be perceived by program leadership is valid. This could exacerbate biases already woven into other means of recognition, promotion and advancement, such as milestones scores [41, 42], faculty [34, 41, 43] and peer evaluations [44], letters of recommendation [45, 46], and training award selection [16, 17].

We acknowledge the limitation of applying a binary construct to sex and gender in our study and recognize these issues are urgent for residents who do not hold traditional gender identities. As such, exploring the experience of gender minorities in GME programs is an important next step and could be facilitated by a larger, multi-institution sample not possible based on our limited sample size in this single institution study. We also acknowledge that sexual orientation, race, ethnicity, disability status and the intersection of these and other factors significantly influence the experience of residents and are not addressed in our study. Through the de-identification process we lost the ability to attribute Hi-5s to an individual resident, thus, it is possible, perhaps likely, a few residents received many Hi-5s and many residents received none which could influence our interpretation of our quantitative results. Finally, content analysis of Hi-5 text would be a valuable follow-up approach that could contribute to growing literature on language used in evaluating residents of different backgrounds.

The gendered experience of our Hi-5 program is important for graduate medical education training programs to consider when implementing recognition strategies for promoting trainee wellness to avoid unknowingly exacerbating gender disparities in the residency experience. Ensuring equitable impact of programs intended to promote wellness, gather interprofessional feedback, and support recognition and advancement is critical for the professional development of all residents.

### Supplementary Information

**Additional file 1:** **Supplementary Table 1. **Characteristics of Hi-5s received by IM residents during study period. Counts are of Hi-5s received, percents represent proportions within each category, with column A representing all Hi-5s, column B those received by female residents and column C those received by male residents. Females comprised 45% of the class size in both 2020-2021 and 2021-2022.

**Additional file 2:** **Supplementary Table 2. **Characteristics of Hi-5s received by pediatric residents during study period. Counts are of Hi-5s received, percents represent proportions within each category, with column A representing all Hi-5s, column B those received by female residents and column C those received by male residents. Females compromised 77% of the 2020-2021 class and 66% of the 2021-2022 class.

**Additional file 3:** **Supplementary Table 3. **Characteristics of Hi-5s received by general surgery residents during study period. Counts are of Hi-5s received, percents represent proportions within each category, with column A representing all Hi-5s, column B those received by female residents and column C those received by male residents. Females accounted for 48% of the 2020-2021 class and 53% of the 2021-2022 class.

## Data Availability

The datasets generated and/or analyzed during the current study are not publicly available due to privacy reasons but are available from the corresponding author on reasonable request.

## Abbreviations

IM: Internal Medicine
GME: Graduate Medical Education
PGY: Post-graduate year
UWSMPH: University of Wisconsin School of Medicine and Public Health
