# Supplementary material for: Gender influences resident physicians’ perception of an employee-to-employee recognition program: a mixed methods study
Source: BMC Med Educ. 2024 Feb 1;24:109. doi: 10.1186/s12909-024-05083-0 (PMC10835820; doi:10.1186/s12909-024-05083-0)
Supplement: Supplementary file 1 — Additional file 1: Supplementary Table 1. Characteristics of Hi-5s received by IM residents during study period. Counts are of Hi-5s received, percents represent proportions within each category, with column A representing all Hi-5s, column B those received by female residents and column C those received by male residents. Females comprised 45% of the class size in both 2020-2021 and 2021-2022. [file 12909_2024_5083_MOESM1_ESM.docx]

| **Supplementary Table 1.** Characteristics of Hi-5s received by IM residents during study period. Counts are of Hi-5s received, percents represent proportions within each category, with column A representing all Hi-5s, column B those received by female residents and column C those received by male residents. Females comprised 45% of the class size in both 2020-2021 and 2021-2022. | | | |
| --- | --- | --- | --- |
| Variable | Total cohort  N = 196 | Female Receiver  N = 99 | Male Receiver  N = 97 |
| Academic year |  |  |  |
| 2020 - 2021 | 89(45.4) | 47(47.5) | 42 (43.3) |
| 2021 - 2022 | 107 (54.6) | 52 (52.5) | 55 (56.7) |
| Receiver PGY |  |  |  |
| PGY1 | 56 (28.6 | 28 (28.3) | 28 (28.9) |
| PGY2 | 72(36.7) | 37 (37.4) | 35 (36.1) |
| PGY3 | 68 (34.7) | 34 (34.3) | 34 (35.1) |
| Sender Role |  |  |  |
| Admin | 6 (3.1) | 4 (4.0) | 2 (2.1) |
| Allied health professionals, other | 7 (3.6) | 2 (2.0) | 5 (5.2) |
| Attending | 33 (16.8) | 15 (15.2) | 18 (18.6) |
| Chief Resident | 70 (35.7) | 34 (34.3) | 36 (37.1) |
| Nurse | 44 (22.4) | 20 (20.2) | 24 (24.7) |
| Trainee | 36 (18.4) | 24 (24.4) | 12 (12.4) |
|  | | | |
