# Supplementary material for: Gender influences resident physicians’ perception of an employee-to-employee recognition program: a mixed methods study
Source: BMC Med Educ. 2024 Feb 1;24:109. doi: 10.1186/s12909-024-05083-0 (PMC10835820; doi:10.1186/s12909-024-05083-0)
Supplement: Supplementary file 2 — Additional file 2: Supplementary Table 2. Characteristics of Hi-5s received by pediatric residents during study period. Counts are of Hi-5s received, percents represent proportions within each category, with column A representing all Hi-5s, column B those received by female residents and column C those received by male residents. Females compromised 77% of the 2020-2021 class and 66% of the 2021-2022 class. [file 12909_2024_5083_MOESM2_ESM.docx]

| **Supplementary Table 2.** Characteristics of Hi-5s received by pediatric residents during study period. Counts are of Hi-5s received, percents represent proportions within each category, with column A representing all Hi-5s, column B those received by female residents and column C those received by male residents. Females compromised 77% of the 2020-2021 class and 66% of the 2021-2022 class. | | | |
| --- | --- | --- | --- |
| Variable | Total cohort  N = 76 | Female Receiver  N = 55 | Male Receiver  N = 21 |
| Academic year |  |  |  |
| 2020 - 2021 | 63 (82.9) | 48 (87.3) | 15 (71.4) |
| 2021 - 2022 | 13 (17.1) | 7 (12.7) | 6 (28.6) |
| Receiver PGY |  |  |  |
| PGY1 | 20 (26.3) | 15 (27.3) | 5 (23.8) |
| PGY2 | 32 (42.1) | 21 (38.2) | 11 (52.4) |
| PGY3 | 24 (31.6) | 19 (34.5) | 5 (23.8 |
| Sender Role |  |  |  |
| Admin | 1 (1.3) | 1 (1.8) | 0 (0) |
| Allied health professionals, other | 12 (15.8) | 8 (14.5) | 3 (14.3) |
| Attending | 12(15.8)) | 12 (21.8) | 1 (4.8) |
| Chief Resident | 3 (3.9) | 2 (3.6) | 1 (4.8) |
| Nurse | 25(32.9) | 17 (30.9) | 8 (38.1) |
| Trainee | 23 (30.3) | 15 (27.3) | 8 (38.1) |
