# Supplementary material for: Gender influences resident physicians’ perception of an employee-to-employee recognition program: a mixed methods study
Source: BMC Med Educ. 2024 Feb 1;24:109. doi: 10.1186/s12909-024-05083-0 (PMC10835820; doi:10.1186/s12909-024-05083-0)
Supplement: Supplementary file 3 — Additional file 3: Supplementary Table 3. Characteristics of Hi-5s received by general surgery residents during study period. Counts are of Hi-5s received, percents represent proportions within each category, with column A representing all Hi-5s, column B those received by female residents and column C those received by male residents. Females accounted for 48% of the 2020-2021 class and 53% of the 2021-2022 class. [file 12909_2024_5083_MOESM3_ESM.docx]

| **Supplementary Table 3.** Characteristics of Hi-5s received by general surgery residents during study period. Counts are of Hi-5s received, percents represent proportions within each category, with column A representing all Hi-5s, column B those received by female residents and column C those received by male residents. Females accounted for 48% of the 2020-2021 class and 53% of the 2021-2022 class. | | | |
| --- | --- | --- | --- |
| Variable | Total cohort  N = 111 | Female Receiver  N = 45 | Male Receiver  N = 65 |
| Academic year |  |  |  |
| 2020 - 2021 | 59 (53.6) | 22 (48.9) | 37 (56.9) |
| 2021 - 2022 | 51 (46.4) | 23 (51.1) | 28 (43.1) |
| Receiver PGY |  |  |  |
| PGY1 | 39 (35.5) | 16 (35.6) | 23(35.4) |
| PGY2 | 33 (30.0) | 8 (17.8) | 25 (38.5) |
| PGY3 | 7 (6.4) | 3 (6.7) | 4 (6.2) |
| PGY4 | 17 (15.5) | 12(26.7) | 5 (7.7) |
| PGY5 | 8 (7.3) | 1 (2.2) | 7 (10.8) |
| Research | 6 (5.5) | 5 (11.1) | 1 (1.5) |
| Sender Role |  |  |  |
| Admin | 8 (7.3) | 6 (13.3) | 2 (3.1) |
| Allied health professionals, other | 11 (10.0) | 3 (6.7) | 8 (12.3) |
| Attending | 28 (25.4) | 14 (31.1) | 14 (21.5) |
| Nurse | 53 (48.2) | 18 (40.0) | 35 (53.9) |
| Trainee | 10 (9.1) | 4 (8.9) | 6 (9.2) |
